# Motivation for COVID-19 Vaccination in Priority Occupational Groups: A Cross-Sectional Survey

**DOI:** 10.3390/ijerph182111726

**Published:** 2021-11-08

**Authors:** Ladislav Štěpánek, Magdaléna Janošíková, Marie Nakládalová, Kateřina Ivanová, Jakub Macík, Alena Boriková, Helena Vildová

**Affiliations:** 1 Department of Occupational Medicine, Faculty of Medicine and Dentistry, University Hospital Olomouc, Palacký University Olomouc, I. P. Pavlova 6, 77900 Olomouc, Czech Republic; magdalena.janosikova@fnol.cz (M.J.); marie.nakladalova@fnol.cz (M.N.); jakub.macik01@upol.cz (J.M.); alena.borikova@fnol.cz (A.B.); helena.vildova@fnol.cz (H.V.); 2 Department of Public Health, Faculty of Medicine and Dentistry, Palacký University Olomouc, Hněvotínská 3, 77515 Olomouc, Czech Republic; katerina.ivanova@upol.cz

**Keywords:** COVID-19, vaccination, motive, occupational group, social service, security forces

## Abstract

Due to the limited availability of COVID-19 vaccines, occupational groups with priority access were identified prior to vaccination. The study aimed to analyze motives for vaccination in these occupational groups. Methods: Members of occupational groups, who were vaccinated at the vaccination center of University Hospital Olomouc before 30 April 2021, were asked to fill in an online questionnaire. Results: A total of 3224 completed questionnaires were obtained from 1332 healthcare workers, 1257 school employees, 363 social service workers, 210 security force members, and 62 critical infrastructure workers. The most frequent motive for vaccination was the effort to protect family members (76.2%), the effort to prevent the spread of COVID-19 in one’s profession (72.3%), followed by concerns about COVID-19 itself (49.1%) and exemptions from anti-epidemic measures (36.8%). Only for social services, the motive focused on one’s profession was mentioned more often (75.2%) than the motive focused on the family (71.1%). At the level of detailed profession-oriented motives, a collegial effort of security force members to protect co-workers and not to endanger the workplace was dominant. Conclusions: The effort to prevent the spread of COVID-19 in the professional environment is a strong motive for vaccination, and strongest among social service workers.

## 1. Introduction

At the end of 2020, vaccinations against the coronavirus disease 2019 (COVID-19) began; however, the supply of vaccines was insufficient due to limited production capacities. As a way of protecting essential services and infrastructure during the ongoing pandemic, indispensable occupational groups were granted priority access to vaccination under relevant vaccination strategies [1,2].

Vaccination in the Czech Republic began in the last days of 2020, initially among healthcare workers (HCWs), who were in close contact with COVID-19 positive patients and seniors. The vaccination of social service workers and outpatient HCWs began around 10 January 2021. Teachers and other school employees gained access to vaccination on 27 February 2021; security force members and critical infrastructure workers on 29 March 2021 [3,4].

The continuing vaccinations against COVID-19 indicate that the availability of vaccines does not automatically translate into an uptake. Vaccination uptake is generally influenced by various factors, including access to and affordability of immunization services, awareness of vaccination, social norms, misinformation, perceptions of vaccines and personal attitudes towards vaccination [5]. The willingness and attitudes towards accepting COVID-19 vaccines are also considerably region-specific [6]. Knowing the underlying motivational causes for getting vaccinated is important for creating effective strategies addressing reluctant individuals (not only from priority groups), which leads to increasing vaccination coverage [7].

The present study aimed to analyze the motivation for accepting vaccination against COVID-19 among priority occupational groups in a robust sample of respondents, especially in the initial period of vaccination. It also focused on presenting a more detailed analysis of the motives associated with a given profession.

### 1.1. Materials and Methods

#### 1.1.1. Study Population

The study population consisted of workers who were voluntarily vaccinated, based on their affiliation with occupational groups with priority access to vaccination in the first months of 2021: HCWs (except for University Hospital Olomouc (UHO) HCWs, who were excluded from this study), school employees, security force members (the police, etc.), social service workers, and critical infrastructure workers (power plant workers, etc.).

Priority occupational groups were identified by the vaccination strategy of the Ministry of Health of the Czech Republic [3]. The study only included individuals (*n* = 7480) who had received at least one dose of the COVID-19 vaccine at the vaccination center of UHO before 30 April 2021, which was the date that these individuals received a questionnaire sent to the personal email address that they had provided prior to their vaccination. In the first months of 2021, the catchment area of the vaccination center of UHO (in terms of the vaccination of all priority occupational groups) included the entire Olomouc district of the Czech Republic (235,000 inhabitants) [8]. The questionnaire was prepared in Google Forms, distributed as a link (URL) sent out by the Department of Communication and Unified Visual Style of UHO, and accompanied with a cover letter providing basic information about the research purpose, ethics approval, and the email addresses and telephone numbers of relevant persons. Both the cover letter and the questionnaire were written in Czech. Data were collected until 30 June 2021. More than 95% of the individuals accepted the Comirnaty vaccine from Pfizer/BioNTech.

#### 1.1.2. Data Analysis

The collected data were statistically analyzed in the Statistica 14 software (TIBCO Software Inc, USA) using methods of descriptive statistics, including chi-square tests and the analysis of variance (ANOVA). The level of statistical significance was set at *p* = 0.05. The Bonferroni correction was applied to adjust for multiple testing to minimize the risk of a type 1 error.

#### 1.1.3. Survey Questionnaire

The survey was conducted using the same questionnaire as in a recent study by the same authors [9]. To meet the aim of the study, closed-ended questions were added to the questionnaire regarding the respondents’ occupation, education, job position (managerial/non-managerial), contact with a large number of persons for most of their time at work (yes/no), and an open-ended question about the number of colleagues they come into close contact with in their profession. To specify the strongest motive for vaccination, a multiple-choice question on motives was followed by a single-choice question on the strongest motive using an identical set of motives in both questions (Table S1). Respondents who chose “the effort to prevent the spread of COVID-19 during the performance of my profession” as the strongest motive in the single-choice question, were further invited to specify detailed motives focused on their profession in another multiple-choice question.

## 2. Results

### 2.1. Characteristics of Survey Respondents

A total of 3224 completed questionnaires were obtained with a response rate of 43.1%. Most completed questionnaires were acquired from HCWs (*n* = 1332) and school workers (*n* = 1257) (Table 1). Among all respondents, women significantly predominated, but in the group of security forces and critical infrastructure, the representation of the sexes was reversed. In terms of common profession characteristics, most respondents had a university degree (58.6%), worked in a non-managerial position (83.5%) and were regularly in contact with a large number of persons for most of their time at work (85.1%).

Security force members stated the highest number of colleagues they were in close contact with in their profession, almost 10. 31% of all respondents identified themselves as chronically ill and 25% of all respondents reported that they had been infected with COVID-19. The highest proportion of vaccinated respondents with a history of COVID-19 was among social service workers (almost 30%), but without a statistical significance of the differences between occupational groups. The lowest level of fear of COVID-19 on a 5-point scale was expressed by members of security forces, while the highest level of fear was shown by vaccinated critical infrastructure workers.

### 2.2. Motivation for Vaccination

Among all respondents, both in the multiple- and single-choice question, the most frequently selected motive for vaccination was the effort to protect family members, followed by the effort to prevent the spread of COVID-19 during professional practice, and then, less often, by concerns about COVID-19 itself and the exemption from restrictive COVID-19 measures after vaccination (Table 2). The difference in how frequently the two most common motives were selected in the multiple-choice question was low; however, it was more pronounced in the single-choice question, where respondents were asked to choose the strongest motive.

At the level of occupational groups, social service workers were the only group with a different order of strongest motives, as they were most motivated by the effort to prevent the spread of COVID-19 in the profession (Table 2). Aside from in respondents from social services, this profession-oriented motive also appeared frequently among HCWs and school employees, although came second after the effort to protect family members. The effort to protect family members was the most motivating factor for security forces. In the multiple-choice question, however, the selection of this motive did not show a statistically significant difference between the occupational groups. Concerns about COVID-19 itself were most often stated as a motive for vaccination by school employees and critical infrastructure workers.

Pairwise comparisons in the whole study sample, regardless of occupational groups, revealed the following statistically significant differences:

Respondents in contact with a large number of persons for most of their time at work more often chose the effort to prevent the spread of COVID-19 in their profession in the multiple-choice question (74% vs. 62.5%, *p* = <0.001).

Respondents without a history of COVID-19 were more motivated by fear of the disease itself both in the multiple- (51.5% vs. 42.4%, *p* < 0.001) and single-choice question (23.3% vs. 16.1%, *p* < 0.001). This motive was also significantly more often selected by the chronically ill compared to the healthy, both in the multiple- (60.7% vs. 43.8%, *p* < 0.001) and single-choice question (28.3% vs. 18.4, *p* < 0.001).

Table 3 shows the distribution of detailed profession-oriented motives among those who identified the effort to prevent the spread of COVID-19 during the practice of their profession as the strongest motive for vaccination in the single-choice question. The most common detailed motive was the effort not to spread the infection to people with whom the respondent worked, followed by a similar effort focused on colleagues and the effort not to endanger the functioning of the workplace in case of work incapacity due to COVID-19. In comparison with other occupational groups, security force members opted significantly more often for the effort to protect colleagues and the workplace, along with the intention to be a positive role model for unvaccinated colleagues.

## 3. Discussion

According to the obtained results, the most frequent motive for vaccination in the whole study sample was the effort to protect family members, followed by the effort to prevent the spread of COVID-19 in the profession, i.e., the desire to protect other people. Among the occupational groups, respondents from social services deviated from this order by being most motivated by the effort to prevent the spread of the infection in their profession. This may be related to differences in this professional sector because social workers, especially those working in institutional care, form interconnected teams of closely cooperating individuals providing long-term care to clients who are seldom changed [10,11]. A part of social work is also professional empathy, a skill that is highly trained in social workers [12]. In our study results, this corresponds to the frequent motivation to get vaccinated to protect close people from the infection, both in the workplace and in the family.

The effort to protect the family from COVID-19 was most prominent among security force members, while the effort to prevent the spread of COVID-19 in the profession was uncommon. However, if it occurred, then it was specified (at the level of detailed motives) as an effort to protect colleagues and the workplace, significantly more often than in other occupational groups. This distinctive result in the security forces group may be associated with the highest average number of colleagues in close contact compared to other groups and also by a prevalent presence of men.

HCWs formed the most numerous occupational group in the present study. A large part of the available research on motives for vaccination against COVID-19 is focused on HCWs. Most studies analyzed the motives before the actual onset of vaccination. A study by Raftopoulos et al., amongst 2238 Greek and Cypriot HCWs, revealed the same order of the strongest motives as in the HCWs from our study, i.e., family protection being the primary motive (98.7%), followed by patient (95.2%) and self-protection (94.2%) [13]. The same order of vaccination motives was also found in a recent study among already vaccinated employees of UHO [9].

Among 346 Italian dentists who intended to accept COVID-19 vaccination, family protection (87%) also dominated as a motive, followed by self-protection (85%), and with patient protection (79%) in third place [14]. The order of the second and third most common motives was reversed in our study, however, with lower percentages in the multiple-choice question. Furthermore, 32% of the dentists in the study by Belingheri et al. agreed to receive a vaccination in order to return to normal activities (such as travelling or attending concerts and celebrations), 30% in order not to miss working days, and 18% to comply with health ministry recommendations. Finally, approximately 8% of the dentists reported wanting to be vaccinated to avoid having to wear masks [14]. Exemptions from anti-epidemic measures were also least frequent in our study.

In a survey by Fakonti et al. (*n* = 437), Cypriot nurses and midwives were willing to accept COVID-19 vaccines because of their work environment (26%), to protect themselves (25.5%), their families (21%), and their patients (15%), as well as the vaccine being provided for free (6.5%) and strongly encouraged in their workplace (6%) [15]. The survey was based on single-choice questions about motivation, unlike the other cited studies. Fear of infecting their family, especially their parents, and becoming infected themselves were the most frequent motives (77.7% and 35.1%, respectively) in a sample of 2133 Egyptian medical students [16].

Similar to the aforementioned study among employees of UHO [9], Kozak et al. performed a survey among 3401 HCWs and social workers, who were already largely vaccinated. Most of them had chosen to be vaccinated to protect their patients, clients, family members (88%), themselves (87%) and the workplace (84%). They also wished to contribute to the further easing of restrictions (80%), enable social contact (75%), and to have more travel opportunities (70%) [17]. This means that, as in the present study and the cited recent study among employees of UHO [9], all of which analyzed the situation of already vaccinated individuals, motives that aimed to protect others were dominant. With regards to the top reasons for COVID-19 vaccination hesitancy in HCWs, the majority of studies found concerns about vaccine safety, efficacy, and potential side effects [18].

The general perception of the COVID-19 vaccine, attitudes among the population and vaccine acceptance, result from a complex combination of demographic, psychological, behavioral, and social factors. A review of 48 articles about the intention to be vaccinated against COVID-19 conducted among the general population found that a person’s age (advanced), sex (male), education level, race/ethnicity, perception of vaccine safety and effectiveness, influenza vaccination history, and self-protection from COVID-19 were all prominent factors associated with the intention to accept the vaccine [18,19].

Certain relatively small differences between occupational groups may be explained by the prioritization of access to vaccination for these groups, because they generally have a higher epidemiological risk of acquiring the disease through more frequent and closer contact with others [20,21]. The occupational groups analyzed in our study enjoy high prestige in the society, also expressed by their ethical standards [22]. Social service workers needed to be in contact with clients even during lockdowns as clients greatly depend on their services, and essential security forces are required to be in the state of constant and continuous readiness. Professional formation, the influence of organizational culture and the characteristics of relevant institutions have a demonstrable effect on the shared values and goals of occupational groups [23,24,25].

Dorman et al. revealed, in their questionnaire survey (*n* = 26,324), that the willingness and reasons to be vaccinated against COVID-19 were statistically significantly and differed among the 11 occupational groups. The construction, maintenance and landscaping workers, homemakers, housekeepers, cleaning and janitorial workers, and retail and food service were least willing to be vaccinated, whereas the retired, students, disabled/unemployed, and HCWs were most willing. Strong predictors of willingness were the confidence in the safety of the vaccine, the intention to protect others by becoming vaccinated, and believing COVID-19 was serious enough to merit vaccination. Protecting others was the strongest motive for 8 out of 11 occupational groups [26]. However, the literature is scarce regarding sources coping with potential profession-related factors influencing attitudes towards vaccination, which limits our options to compare results. Due to their irreplaceability in the society, it is necessary to also focus research also on other priority occupational groups besides HCWs.

Experience with other well-established vaccination programs can also provide guidance for COVID-19 vaccination, e.g., the motivation for the seasonal influenza vaccination (SIV) has been studied for a long time, but mainly among HCWs. Self-protection was the most common motive for SIV in a country-wide study among 27,163 German HCWs from 171 hospitals in the influenza season 2018/2019 [27], as well as among HCWs of UHO in the season 2020/2021 [28]. In the case of vaccination against COVID-19, self-protection appears to be a weaker motive now then during the pandemic.

A limitation of the study is unknown response rates in particular occupational groups, as the criterion for including occupational groups in the study was only their priority access to vaccination, without any preliminary knowledge of the specific group.

Although the catchment area of the vaccination center of UHO, in terms of the vaccination of all priority occupational groups, included the entire Olomouc district. HCWs from bigger healthcare facilities and security force members, especially members of the army, may have been vaccinated elsewhere. Therefore, these two occupational groups may not be population-representative.

## 4. Conclusions

Among the priority occupational groups, the strongest motive for vaccination was the effort to protect family members, followed by the effort to prevent the spread of COVID-19 in the profession. In social service workers, the order of strongest motives was reversed, i.e., the effort to prevent the spread of the infection during the performance of the profession dominated. In security force members, compared to other occupational groups, profession-oriented motives were tied to a collegial effort to protect co-workers and not to endanger the functioning of the workplace. The results of the study may serve as a tool for strategies aimed at increasing the motivation to become vaccinated against COVID-19, and not only in priority occupational groups.

## Figures and Tables

**Table 1:** Occupational groups’ characteristics.

Characteristics	Social Services	Security Forces	Healthcare Workers	Critical Infrastructure	School Employees
Respondents (*n*)	363	210	1332	62	1257
Sex-females (*n*, %) *	305 (84%)	46 (21.9%)	1104 (82.9%)	16 (25.8%)	1022 (81.3%)
Age (average ± SD)	43.1 ± 10.5	42.9 ± 9.2	41.0 ± 10.3	48.2 ± 8.5	45.9 ± 9.4
Education *					
Primary (*n*, %)	6 (1.7%)	0	7 (0.5%)	1 (1.6%)	3 (0.2%)
Lower secondary (*n*, %)	55 (15.2%)	0	36 (2.7%)	6 (9.7%)	71 (5.6%)
Upper secondary (*n*, %)	148 (40.8%)	86 (41%)	573 (43%)	39 (62.9%)	291 (23.2%)
University (*n*, %)	153 (42.1%)	124 (59%)	711 (53.4%)	15 (24.2%)	887 (70.6%)
Managerial job position (*n*, %) *	82 (22.6%)	44 (21%)	179 (13.4%)	11 (17.7%)	217 (17.3%)
Contact with a large number of persons at work (*n*, %) *	289 (79.6%)	148 (70.5%)	1149 (86.3%)	31 (50%)	1129 (89.8%)
Number of colleagues in close contact in the profession (average ± SD)	7.5 ± 6.5	9.6 ± 12.2	7 ± 7.4	6 ± 7.1	8.9 ± 13.7
With a chronic disease (*n*, %) *	118 (32.5%)	47 (22.4%)	404 (30.3%)	37 (59.7%)	396 (31.5%)
History of COVID-19 (*n*, %)	108 (29.8%)	58 (27.6%)	345 (25.9%)	14 (22.6%)	295 (23.5%)
Level of fear of COVID-19 (average ± SD)	2.7 ± 1.2	2.4 ± 1.1	2.6 ± 1.1	2.9 ± 1.3	2.7 ± 1.2

SD, standard deviation, * statistically significant differences among occupational groups.

**Table 2:** Motives for vaccination against COVID-19 and the frequency of their selection.

Occupational Groups	Concerns about COVID-19 Itself	An Effort to Prevent the Spread of COVID-19 During the Performance of My Profession	An Effort to Protect Family Members	Being Exempted from Restrictive COVID-19 Measures after Vaccination
**Multiple-choice question**				
All respondents	1584 (49.1%) *	2332 (72.3%) *	2456 (76.2%)	1188 (36.8%) *
Social services	156 (43%)	273 (75.2%)	258 (71.1%)	136 (37.5%)
Security forces	93 (44.1%)	134 (63.5%)	164 (77.7%)	76 (36%)
Healthcare workers	613 (46%)	973 (73%)	1032 (77.5%)	527 (39.6%)
Critical infrastructure	36 (58.1%)	33 (53.2%)	47 (75.8%)	16 (25.8%)
School employees	686 (54.6%)	919 (71.1%)	955 (76%)	433 (34.4%)
**Single-choice question (strongest motive)**				
All respondents	693 (21.5%) *	789 (24.5%)*	1240 (38.4%) *	385 (11.9%) *
Social services	70 (19.3%)	140 (38.6%)	97 (26.7%)	44 (12.1%)
Security forces	36 (17.1%)	24 (11.4%)	108 (51.2%)	31 (14.7%)
Healthcare workers	254 (19.1%)	316 (23.7%)	530 (39.8%)	177 (13.3%)
Critical infrastructure	23 (37.1%)	6 (9.7%)	23 (37.1%)	6 (9.7%)
School employees	310 (24.7%)	303 (24.1%)	482 (38.3%)	127 (10.1%)

* statistically significant differences among occupational groups.

**Table 3:** Detailed profession-oriented motives for vaccination (multiple-choice).

Occupational Groups	An effort to Prevent the Spread of COVID-19 to People Who I Work with (Clients, Patients, etc.)	An Effort to Prevent the Spread of COVID-19 to Colleagues	An Effort Not to Endanger the Functioning of My Workplace in Case of My Incapacity to Work	Being a Positive Role Model for Unvaccinated Colleagues	An Effort to Prevent a Negative Impact of the Disease on my Professional Development
All respondents	678 (85.9%)	459 (58.2%) *	448 (56.8%) *	210 (26.6%) *	126 (16%) *
Social services	122 (87.1%)	74 (52.9%)	79 (56.4%)	42 (30%)	23 (16.4%)
Security forces	21 (87.5%)	21 (87.5%)	17 (70.8%)	9 (37.5%)	7 (29.2%)
Healthcare workers	271 (86.3%)	182 (58%)	186 (59.2%)	89 (28.3%)	51 (16.2%)
Critical infrastructure	4 (66.7%)	3 (50%)	2 (33.3%)	1 (16.7%)	2 (33.3%)
School employees	260 (86.4%)	179 (59.5%)	164 (54.5%)	69 (22.9%)	43 (14.3%)

* statistically significant differences among occupational groups.

## Data Availability

The data presented in this study are available from the corresponding author upon reasonable request.

## Supplementary Materials

The following are available online at https://www.mdpi.com/article/10.3390/ijerph182111726/s1, Table S1: The questionnaire.
