# Supplementary material for: Motivation for COVID-19 Vaccination in Priority Occupational Groups: A Cross-Sectional Survey
Source: Int J Environ Res Public Health. 2021 Nov 8;18(21):11726. doi: 10.3390/ijerph182111726 (PMC8583662; doi:10.3390/ijerph182111726)
Supplement: Supplementary file 1 [file ijerph-18-11726-s001.zip › ijerph-1418677-supplementary.pdf]

Motivation to COVID-19 vaccination in priority occupational groups: a cross-sectional survey – the questionnaire

Sex:

- male
- female

Age (years): …

To which occupational group with priority access to vaccination against COVID-19 do you belong?

- social services
- security forces
- healthcare workers
- critical infrastructure
- school employees
- other:

What is your highest reached education??

- primary
- lower secondary
- upper secondary
- tertiary/university
- other:

Is your job position mostly managerial?

- yes
- no

Are you in contact with a large number of any persons for most of your time at work?

- yes
- no

How many colleagues are you in close contact with during the performance of your profession? …

Do you suffer from a chronic disease?

- yes
- no

If you suffer from a chronic disease, what is it?

- cardiovascular disease
- arterial hypertension
- kidney disease
- liver disease
- respiratory tract disease
- immune system disease
- diabetes mellitus
- endocrine system disease
- anaemia
- other:

Have you undergone COVID-19?

- yes

- no

Try to assess your overall fear of COVID-19 (its course, consequences...) on a scale of 1-5:

1 – I am not afraid of COVID-19; 5 – I have serious concerns about COVID-19

I was motivated to get vaccinated against COVID-19 by (choose any number of options):

- Concerns about COVID-19 itself
- An effort to prevent the spread of COVID-19 during the performance of my profession
- An effort to protect family members
- Being exempted from restrictive COVID-19 measures after vaccination
- Other:

I was motivated to get vaccinated against COVID-19 by (choose one option/the strongest motive):

- Concerns about COVID-19 itself
- **An effort to prevent the spread of COVID-19 during the performance of my profession**
- An effort to protect family members
- Being exempted from restrictive COVID-19 measures after vaccination
- Other:

For those, who chose the option highlighted in bold

In terms of the effort to prevent the spread of COVID-19 in the profession, I was motivated by (choose any number of detailed options):

- An effort to prevent the spread of COVID-19 to people who I work with (clients, patients, etc.)
- An effort to prevent the spread of COVID-19 to colleagues
- An effort not to endanger the functioning of my workplace in case of my incapacity to work
- Being a positive role model for unvaccinated colleagues
- An effort to prevent a negative impact of the disease on my professional development
- Other:
